# Passive Immunization against HIV/AIDS by Antibody Gene Transfer

**DOI:** 10.3390/v6020428

**Published:** 2014-01-27

**Authors:** Lili Yang, Pin Wang

**Affiliations:** 1 Department of Microbiology, Immunology and Molecular Genetics, Eli & Edythe Broad Center of Regenerative Medicine and Stem Cell Research, University of California at Los Angeles, Los Angeles, CA 90095, USA; 2 Mork Family Department of Chemical Engineering and Materials Science, University of Southern California, Los Angeles, CA 90089, USA; E-Mail: pinwang@usc.edu; 3 Department of Biomedical Engineering, University of Southern California, Los Angeles, CA 90089, USA; 4 Department of Pharmacology and Pharmaceutical Sciences, University of Southern California, Los Angeles, CA 90089, USA

**Keywords:** antibody gene transfer, human immunodeficiency virus, vectored immunoprophylaxis, broadly neutralizing antibody, adeno-associated virus-based vectors

## Abstract

Despite tremendous efforts over the course of many years, the quest for an effective HIV vaccine by the classical method of active immunization remains largely elusive. However, two recent studies in mice and macaques have now demonstrated a new strategy designated as Vectored ImmunoProphylaxis (VIP), which involves passive immunization by viral vector-mediated delivery of genes encoding broadly neutralizing antibodies (bnAbs) for *in vivo* expression. Robust protection against virus infection was observed in preclinical settings when animals were given VIP to express monoclonal neutralizing antibodies. This unorthodox approach raises new promise for combating the ongoing global HIV pandemic. In this article, we survey the status of antibody gene transfer, review the revolutionary progress on isolation of extremely bnAbs, detail VIP experiments against HIV and its related virus conduced in humanized mice and macaque monkeys, and discuss the pros and cons of VIP and its opportunities and challenges towards clinical applications to control HIV/AIDS endemics.

## 1. Introduction

Since the emergence of Acquired Immune Deficiency Syndrome (AIDS) more than 30 years ago, over 25 million people have died of AIDS, and about 34.2 million have been infected with human immunodeficiency virus (HIV), the virus that causes AIDS [1,2]. The disease has a disproportionately larger impact in underdeveloped parts of the world, where AIDS has devastated whole countries, especially in Africa, killing both adults and children and dramatically decreasing life expectancy and economic growth. Although the development of drug-based anti-retroviral therapy (ART) has slowed, or even halted, the progression of AIDS, it cannot cure the disease; for most cases, HIV-infected individuals left untreated do not survive. This lifelong dependence on drug therapy raises significant concerns on the sustainability and affordability of ART and presents daunting global economic and health problems [2]. 

It is widely agreed that the most effective method to stop or slow the AIDS epidemic is a safe and efficacious vaccine [3,4,5]. Unfortunately, despite almost 30 years of intense scientific investigations, no effective HIV/AIDS vaccine is approaching licensure. To account for such failure, scientists have pointed to the unique properties that HIV has evolved to evade immune recognition [6,7]. Accumulating evidence from other viruses and HIV-related animal model studies suggests the need for vaccine-elicited neutralizing antibodies (nAbs) as the most effective protection against HIV infection [8,9,10,11,12,13,14,15,16]. However, HIV is an enveloped retrovirus that presents challenges for conventional nAb-based vaccine strategies. The virus mutates rapidly to change its surface structure, utilizes host-derived nonimmunogenic glycans to mask its exposed surface, and hides its conserved and potentially vulnerable regions, such as the CD4 binding site in the interfaces of oligomeric proteins [6,17]. Although 20% of chronically HIV-infected individuals generated nAbs and 2%–4% of them have broadly neutralizing antibodies (bnAbs) capable of neutralizing most tested HIV strains [18], these antibodies are only produced after months to years of virus infection [1]. The challenge lies in identifying vaccine preparations and delivery methods to elicit antibodies, preferably nAbs, to protect humans from infection upon HIV exposure. 

**Figure 1:**
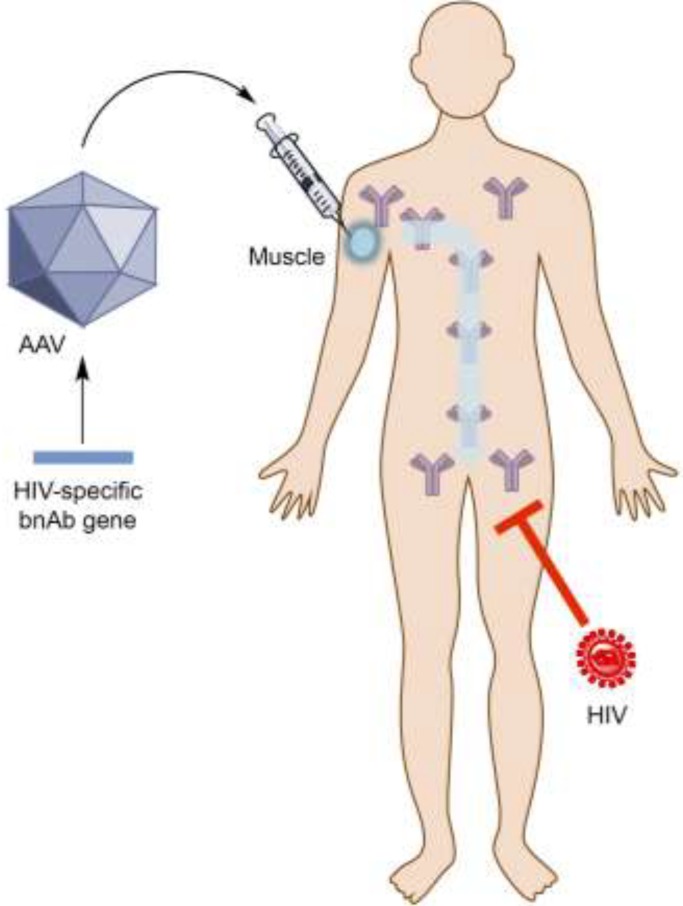
Schematic representation of the Adeno-associated virus-based vector (AAV)-based Vectored ImmunoProphylaxis (VIP) approach against human immunodeficiency virus (HIV).

As an alternative to traditional vaccine methods, recent studies in mice [19] and monkeys [20] demonstrated a gene therapy approach for generating vaccine-like protection by delivering genes encoding nAbs into nonhematopoietic tissues, such as muscles [21,22,23]. This novel strategy is called Vectored ImmunoProphylaxis (VIP) (Figure 1). The advantage of this approach lies in the direct provision of nAbs through transgene expression in host cells, bypassing the reliance on the natural immune system for mounting desired humoral immune responses. VIP extends the application of monoclonal antibodies (mAbs) from passive immunization to a new form of gene therapy that is based on transfer of antibody genes and their subsequent expression in host tissues. In this article, we will review antibody-based gene transfer and the recent development of HIV-specific bnAbs, followed by a discussion of the pros and cons of VIP and its opportunities and challenges towards clinical applications to control HIV/AIDS endemics. 

## 2. Antibody Gene Transfer

Since 1986, the US Food and Drug Administration (FDA) has so far approved nearly 30 monoclonal antibodies (mAbs) as therapeutic drugs for treating patients with cancer and with autoimmune, inflammatory and infectious diseases [24,25]. Many more promising mAbs are in preclinical and clinical development. Thus, therapeutic antibodies have become the fastest growing class of therapeutic molecules in the pharmaceutical industry [26,27,28]. Antibody therapies generally involve high doses over a long period of time, thereby requiring large amounts of clinical-grade reagents for treating one patient, and yet mAbs are among the most complicated and expensive pharmaceutical products to manufacture [29,30,31,32,33]. Therefore, development of robust manufacturing processes to produce individual mAbs with high capacity and yield remains a bottleneck for rapid delivery of therapeutic benefits to patients. To overcome this barrier, an alternative approach would involve the body itself in completing antibody production. Indeed, a myriad of preclinical studies have demonstrated that antibody production *in vivo* after gene transfer is feasible and that it can potentially accelerate the translation of therapeutic mAbs from bench to bedside. 

### 2.1. Delivery Methods

A key step in antibody gene transfer is the identification of appropriate delivery vectors to efficiently deliver antibody genes into host tissues for *in vivo* expression. Nonviral delivery by electroporation of naked DNA to muscle cells has been explored for *in vivo* transfer of genes encoding mAbs [34,35,36,37]. Plasma antibody concentrations of 0.4–1.5 μg/mL were observed in mice and sheep for a period of 6–7 months. This confirmed that skeletal muscle cells possess the cellular factors required to synthesize antibodies and that highly vascularized muscle can transport produced antibodies into the systemic circulation. Although nonviral vectors are easy to produce and do not induce vector-specific immune responses [38], the transfer efficiency is low, yielding only minimal production of antibodies, which may not be practical for therapy. 

Based on their higher transduction efficiency, various viral vectors have been tested for *in vivo* antibody gene transfer. Intravenous administration of adenoviral vectors (Ads) to mice showed long-term average antibody concentrations in serum ranging from ~0.02 μg/mL to >40 μg/mL, with peak concentrations as high as 1 mg/mL [39,40,41]. These experiments used the most well-studied vector derived from serotype 5 of human adenovirus (Ad5). High transgene expression usually occurred in liver, lung and spleen for this vector after tail vein injection in mice. Antibody production could be detected as early as day 1 and peaked at days 3–6 post-administration, after which expression decreased rather quickly [41]. In addition to its transient expression, which is not suited for antibody therapy requiring sustained delivery, clinical utilization of Ads is hindered by the inflammatory and immune response they evoked after *in vivo* administration [38,42,43]. Many Ad-based gene therapy studies concluded that this vector system might be best suited for applications that need only transient expression and that immune stimulation is desired, such as genetic vaccination and cancer gene therapy [44]. 

Adeno-associated virus-based vectors (AAVs), while not integrated into the genome, can transduce nonreplicating and long-lived cells *in vivo*, and the resulting expression can persist for months to years in their associated tissues [38,44]. This also indicates that anti-vector immunity induced by AAVs is at a manageable level [45] so that vector-modified cells can survive from the immune system for a long period of time. Thus, various serotypes of AAVs have become the most appealing vectors for *in vivo* gene therapy [46], and many of them have shown promising results in early-phase clinical trials [47,48,49,50,51,52,53,54,55,56,57,58]. The challenge for AAVs as delivery vehicles is their limited packaging capacity. Therefore, since AAVs cannot accommodate the conventional antibody expression cassette with heavy and light chains under two separate promoters or with heavy and light chains under one promoter but linked by a DNA sequence for internal ribosome entry site (IRES). Clark and coworkers constructed a dual-promoter AAV2 for expression of IgG1b12 antibody [59]. Although the vector could be produced, its titer was compromised owing to the large DNA insert that reached the packaging limit of AAV2. As a compensatory method, the same group had to design an immunoadhesin form of antibody-like molecules, which has shorter sequences, for efficient packaging and production of high quality AAVs [20]. However, this packaging barrier has now been overcome by an elegant approach, in which a foot-and-mouth-disease virus (FMDV)-derived 2A self-processing sequence (F2A, only 72 base pairs long) is engineered with a furin cleavage site. Once placed between heavy and light chains, the efficient expression of antibodies by AAVs is enabled from a single reading frame driven by one promoter [60]. The *in vivo* administration of AAV8 (4 × 10^11^ genome copies (gc)) with this FMDV 2A configuration through the hepatic portal vein led to average serum antibody of ~1 mg/mL for 4 months with peak concentration as high as >8 mg/mL. This work inspired many more studies with various serotypes of AAV administered by the intravenous [61], intranasal [62], intravitreal [63], intrapleural [64] or intramuscular [19] routes for *in vivo* antibody expression. These studies consistently showed high titer antibody production for a long period of time. Thus, it seems that AAVs are the most favorable delivery vectors to achieve durable and efficient *in vivo* expression of antibodies. It should be noted that AAV-mediated antibody expression usually takes two weeks in order to reach a significant level [65], which prevents its application in situations that require immediate antibody delivery. In light of different kinetics of antibody expression mediated by Ads (rapid, but short; 1 day to 4 weeks) and AAVs (slower, but lasting, from two weeks to years), Boyer and coworkers demonstrated that co-administration of Ad and AAV vectors effectively leads to both rapid and sustained delivery of antibodies *in vivo* [65]. 

### 2.2. Diseases Targeted by Antibody Gene Transfer

Passive immunization by therapeutic mAbs has already provided therapeutic benefits for cancer patients [25]. Following this success, antibody gene transfer was tested in various preclinical tumor models [66,67]. Based on their pioneering strategy of using 2A to link antibody heavy and light chains, Jooss and coworkers reported substantially retarded tumor growth after intravenous injection of AAV8 encoding anti-VEGFR2 antibody in both mouse melanoma and human glioma models [60]. Qian and coworkers evaluated the Ad5-mediated intravenous delivery of trastuzumab and found that such treatment could lead to significant elimination of ovarian cancer in mice [40]. The same group also injected Ad5 vectors encoding the Rituxan version of anti-CD20 antibody and observed >40 μg/mL antibody production in serum, which enabled a complete elimination of transplanted B-cell lymphoma in nude mice [68]. In an A431 xenograft tumor model, Hermiston and coworkers were able to show significant inhibition of tumor growth when the anti-EGFR antibody-expressing AAV1 was administered intramuscularly in both prophylactic and therapeutic settings [69]. When a lentiviral vector (LV) was employed to deliver anti-Met antibody via either intravenous or intratumoral route, Comoglio and coworkers reported substantial tumor inhibition in a human colorectal carcinoma xenograft model. Using a similar LV vector, Zheng and coworkers showed that the vector-mediated delivery of anti-DR5 antibody via intramuscular administration prolonged the lifespan of mice inoculated with orthotopic human lung tumor cells [70]. Thus, in addition to Ad and AAV vectors, these two studies also suggest that the LV system can be exploited for viral vector-mediated delivery of antibodies *in vivo* as well. 

The efficacy of antibody gene transfer has also been evaluated in infectious diseases. In addition to HIV, which is discussed later, genetic delivery of the antibody has been used to prevent and/or treat infections induced by respiratory syncytial virus (RSV), the bacteria *Yersinia pestis* and anthrax, and the influenza virus. Worgall and coworkers constructed Ad5 and non-human primate serotype rh.10 AAV (AAVrh.10) vectors encoding palivizumab, an anti-RSV antibody that has been clinically approved to prevent RSV infection in high-risk populations [71,72,73], and tested their ability to prevent RSV infection in mice [64]. While the intravenous injection of Ad5 could reduce RSV infection by at least 5.4-fold, the intrapleural injection of AAVrh.10 was superior, being able to ameliorate infection with 10.6~14.3-fold lower RSV titer. In a lethal *Yersinia pestis* challenge in mice, Boyer and coworkers were able to protect 80% of animals from infection after administration of Ad5 encoding antibody against the V antigen of *Yersinia pestis* [41]. In an anthrax toxin challenge experiment in mice, the same research team demonstrated a dual-component delivery method by co-administration of Ad5 and AAVrh.10 vectors to afford both immediate (by Ad5) and durable (by AAVrh.10) protection [65], which can be important for applications against biowarfare/bioterrorism agents [74]. Chiba and coworkers evaluated an electroporation-based delivery of naked DNA encoding an anti-hemagglutinin antibody and found that the produced antibodies were sufficient to protect mice from lethal influenza virus challenge, despite its relatively transient expression [37]. Subsequently, Baltimore and coworkers demonstrated that intramuscular administration of AAV8 encoding broadly neutralizing antibodies could protect mice from infection by diverse strains of H1N1 influenza [75]. Additionally, Wilson and coworkers showed that intranasal delivery of AAV9 harboring a different broadly neutralizing antibody could elicit broad protection against clinical isolates of H5N1 and H1N1 influenza viruses in mice and ferrets [62]. 

To address cocaine addiction, Crystal and coworkers intravenously administered AAVrh.10 expressing an anti-cocaine antibody into mice and found that the produced antibody was sufficient to sequester intravenously administered cocaine in the blood [76]. Consequently, treated animals were completely resistant to the cocaine. When an anti-nicotine antibody was constructed into AAVrh.10, the same team demonstrated that vector injection yielded nicotine-specific antibodies capable of shielding the brain from systemically administered nicotine, resulting in the absence of physiologic effects of nicotine in the treated mice [61]. These studies expanded the applications of antibody gene transfer from cancer and infectious diseases to addiction disorders, demonstrating the broad utility of this method. 

## 3. Discovery of HIV-Specific Neutralizing Antibodies

For all available human and veterinary vaccines against infectious viruses, the serum level of nAbs correlates closely with the degree of protection [8]. The mechanism of neutralization by nAbs lies in either blocking the interaction between viral envelopes with their receptors or inhibiting viruses for their further transport to cytoplasm [77,78]. Mounting evidence suggests that antibody-based neutralization is also vital for HIV protection [9,10,11,12,13,14,15,16], although antibody-dependent cytotoxicity (ADCC) and antibody-dependent cell-mediated virus inhibition (ADCVI) may play roles in curbing viral load through controlling the later phase of virus replication [79]. Because HIV possesses many features to evade humoral immune responses, many difficulties have arisen in previous years in isolating bnAbs. Before 2009, we had only a limited number of so-called “first-generation bnAbs against HIV” [6,78,80]. However, since 2009, breakthroughs have been made, and a large panel of “second-generation bnAbs against HIV” has been identified. These bnAbs have greater neutralization potency and breadth [78,81], and they are all excellent resources for antibody gene transfer to target HIV. 

### 3.1. First-Generation bnAbs against HIV

The first broadly neutralizing human Ab against HIV is b12, which was isolated from a clade B-infected patient through phage screening of libraries of heavy and light chains [82]. Because of this isolation method, it is unknown whether or not this is a naturally occurring anti-HIV antibody. It was shown that b12 could recognize the CD4 binding site (CD4bs) on the gp120 surface of the HIV envelope (Env) [83] and could neutralize nearly 50% of clade B viral strains and 30% of non-clade B strains [84,85]. Another conserved vulnerable site on the gp120 of the HIV Env is the oligomeric glycan structure on the carbohydrate-masked surface of gp120. One bnAb, 2G12, was determined to bind to this glycan epitope and could neutralize many clade B-derived viral strains [86,87]. Once Env binds its receptor CD4, it triggers conformation changes to form and display a CD4-induced (CD4i) epitope, making it possible to develop HIV-specific mAbs [88]. Although this CD4i epitope is highly conserved among different clades, isolated mAbs, such as 17b, have only weak neutralization reactivity, and their potency can only be boosted if low concentrations of soluble CD4 (CD4s) are added or the Fab form of 17b is used in the neutralization assay [89]. In addition, one region on the gp41 of the HIV Env, called the membrane proximal external region (MPER), has been demonstrated as a good target for HIV-specific nAbs. 2F5 and 4E10 are the two most studied bnAbs that bind both the gp41 MPER and its nearby lipids [90,91]. One of them, 4E10, displays an impressive breadth of neutralization across several clades, although it only has weak to modest potency [92]. Thus, in spite of many years of study (from 1994 to 2008), only a handful of bnAbs (b12, 2G12, 2F5, 4E10) have thus far been identified as first-generation HIV-specific nAbs able to recognize three defined neutralization epitopes: CD4bs, glycan domain, and MPER [93]. 

### 3.2. Second-Generation bnAbs against HIV

Two seminal papers published in 2009 set a new course for discovery of the newer generation bnAbs against HIV [94,95]. To combine the cell sorting of gp140-binding B cells and single-cell antibody cloning, Nussenzweig and coworkers cloned over 500 mAbs to reveal that multiple antibodies targeting a range of Env epitopes contribute to the broad neutralizing serological activity of elite neutralizer in patients infected with HIV [94]. Concurrently, Burton and coworkers utilized a clonal B cell culture to screen over 30,000 activated memory B cells from a clade-A-infected African donor and identified two potent bnAbs, PG9 and PG16 [95], that target the V1/V2 loops of the gp120 [96]. Soon after, HJ16, a bnAb targeting CD4bs, was identified and is capable of neutralizing 40% of viral isolates [97]. Subsequently, the design of antigenically resurfaced glycoproteins with exposed CD4bs led to more successful isolation of CD4bs-directed bnAbs [98]. After screening of several sera, sorting and single-cell cloning were performed on B cells from a donor with neutralizing reactivity. The isolated bnAb, VRC01 [98], could bind to CD4bs [99] and neutralize over 90% of tested HIV strains [98,100]. Building upon their previous work [95], Burton and coworkers screened memory B cells from four elite neutralizers and identified several glycan-dependent bnAbs, designated PGT121-PGT145 [101], some of which can penetrate the glycan shield and recognize an epitope in the vicinity of the V3 loop region, as well as two conserved glycans on gp120 [102]. Further studies isolated substantially more CD4bs-directed bnAbs and improved our understanding of the various mechanisms used by these bnAbs to target HIV through this CD4bs [103,104]. A structural study of one of these bnAbs, NIH45-46, revealed a unique binding mode contributing to the increased interaction between the antibody and gp120, and a single amino acid substitution at this key binding area resulted in a variant, NIH-45-46^G54W^, with markedly enhanced breadth and potency [105]. More rational designs of this variant yielded a mutant, 45-46m2, which has greater breadth, and another mutant, 45-46m7, is capable of targeting a common HIV escape pathway [106]. Substantial advances were also made on MPER-specific antibodies, and an example of such newly identified bnAb, 10E8, could neutralize ~98% of tested virus strains, while at the same time having few of the shortcomings exhibited by previous bnAbs (e.g., 4E10 and 2F5) in this category, such as lipid binding and autoreactivity [107]. Kwong and coworkers have recently provided the most updated and comprehensive list of these HIV-specific bnAbs by clustering them into key recognition epitopes: CD4bs, V1/V2 loop, glycan-V3 site, and MPER [81]. The discovery of these bnAbs is not only transforming our understanding of humoral immune responses against HIV and facilitating the design of immunogens to elicit these antibodies, but it is also enabling researchers to test anti-HIV strategies that depend on passive immunization delivered by antibodies or antibody-encoded genes. 

## 4. Vectored Immunoprophylaxis for HIV by Antibody Gene Transfer

Despite the tremendous success in identifying and characterizing bnAbs against HIV, a formidable challenge remains in designing appropriate forms of immunogens to elicit these antibodies through vaccination [3,78,108]. Passive immunization by antibody infusion showed promising results for HIV protection in macaque monkeys, but this type of long-term repeated treatment is too expensive, making it impractical for broad implementation in humans. Thus, vectored immunoprophylaxis (VIP), which involves a single injection of AAVs for *in vivo* delivery of genes encoding bnAbs, is an attractive alternative and enables continuous and sustained delivery of antibodies to prevent HIV infection. Two recent studies in animal models have demonstrated that VIP against HIV is feasible [19,20], paving the way for further clinical testing in humans. 

### 4.1. VIP against HIV in Mice

To test VIP against HIV in mice, Baltimore and coworkers at the California Institute of Technology constructed AAV8 vectors encoding the full-length of one of bnAbs (b12, 2G12, 4E10, 2F5 and VRC01) [19]. In a pilot test, it was found that a single injection of AAV8 could achieve peak antibody production in serum at week 6. The expression decreased by 2- to 3-fold in the following weeks, but was then stably maintained for the duration of the study (64 weeks). Although the long-term expression was encouraging, the 4E10 antibody titer in serum is modest (~5 μg/mL) and therefore might not be sufficient for HIV protection. A series of optimizations were carried out to develop an improved antibody expression configuration in muscles. After testing several promoters, the authors created a new hybrid promoter, designated CASI, which combined the enhancer of the cytomegalovirus immediate early promoter (CMV), the β-actin promoter, and the enhancer of ubiquitin C (UBC) promoter flanked by a splicing donor and acceptor. *In vivo* expression analysis confirmed that CASI performed better than the conventional promoters tested. Incorporation of the woodchuck hepatitis virus posttranslational regulatory element (WPRE) [109] improved *in vivo* expression of luciferase nearly 10-fold. The authors also optimized the configuration of the antibody transgene by codon-optimization of F2A, replacing the endogenous signal sequences with codon-optimized sequences derived from human growth hormone and removing the potential of inappropriate splicing sequences [110]. The final optimized AAV8 vector could mediate *in vivo* delivery of antibody b12 at a level that was 100-fold higher than that of non-optimized vector after a single injection of the gastrocnemius muscle [19]. 

With the optimal vector in hand, the authors administered immunodeficient mice (NOD/SCID/γc strain, or NSG) with bnAb-encoded AAV8. When stable antibody production was reached (6 weeks), NSG mice were transplanted with human peripheral blood mononuclear cells (PBMCs). After engraftment for 2 weeks, mice were challenged with intravenous injection of the NL4-3 strain of HIV and monitored by the extent of CD4 depletion and the level of HIV p24 in tissues. Among the first-generation bnAbs, b12 (over 100 μg/mL) was the only antibody that afforded full protection, whereas 2G12 (~250 μg/mL), 4E10 (~25 μg/mL), and 2F5 (~25 μg/mL) yielded partial protection. In fact, AAV8-mediated expression of b12 was so potent that it could protect against HIV challenge at doses (125 ng) 100-fold higher than necessary to deplete CD4 T cells in control animals without b12 expression. The authors also tested the delivery of a second-generation bnAb, VRC01, by AAV8 in the same animal model and compared its potency with b12. Although their expression was similar, VRC01 could provide full protection against HIV challenge (10 ng) at a titer of 8.3 μg/mL, while a higher titer of b12 (34 μg/mL) was needed for equal protection, demonstrating *in vivo* that VRC01 is a superior antibody for AAV-mediated gene transfer to fight against HIV. 

Since the publication of this work, the Baltimore team has performed more experiments, and some of the most recent results were presented in a satellite symposium at the AIDS Vaccine 2012 Conference [23]. Most human infections by HIV are through the mucosal surface; consequently, HIV needs to transport across many mucosal barriers for a successful infection [111]. New studies indicated that initial infection usually involves only one or a handful of founder viruses [112,113,114]. Baltimore and colleagues performed VIP to mice to express b12 or VRC01, followed by intravenous HIV challenge with the REJO.c transmitted founder strain. It was observed that VRC01, but not b12, could provide substantial protection. This study not only confirms the superiority of VRC01, but also demonstrates that (1) founder viruses are not resistant to neutralization and (2) the newer generation of bnAbs can neutralize them *in vivo*. In order to model HIV transmission more closely to humans, the Baltimore group adapted a humanized BLT (bone marrow-liver-thymic chimeras) mouse system for VIP testing, in which a low dose, intravaginal challenge of HIV could be implemented. The BLT mice were highly resistant to the intravaginal challenge of a laboratory HIV strain, JR-CSF, after mice were given VIP to express VRC01 or an engineered, more potent VRC01-like antibody, VRC07^G54W^. Despite 15 repetitive exposures to the virus, 5 out of 8 VRC01-expressing mice and 12 out of 12 VRC07^G54W^-expressing mice had no detectable level of HIV. For the two infected mice, the infection occurred at a much later phase of the repetitive challenge. More striking results were observed when the REJO.c transmitted founder strain was used to intravaginally infect mice given VIP to express VRC07^G54W^. All mice were protected with no detectable viral load by a PCR-based viral load assay. This series of experiments on BLT mice demonstrate that VIP can afford protection from mucosal HIV transmission and that VIP can allow for the delivery of not only natural antibodies, but also engineered antibodies, such as VRC07^G54W^.

### 4.2. VIP against SIV in Macaques

Prior to work by the Baltimore team, Johnson and coworkers at the Children’s Hospital of Philadelphia had demonstrated the concept of VIP against simian immunodeficiency virus (SIV), an equivalent of HIV in humans, in macaque monkeys [20]. Without the optimal vector that the Baltimore team created, the Johnson team used the self-complementary AAV (scAAV) vectors with double-stranded genomes for better protein expression *in vivo* [115]. However, the tradeoff of scAAV is the reduced coding capacity for transgenes, making it impossible to carry the full-length of antibody genes. Thus, a designer molecule, called immunoadhesin, which has a shorter sequence than a full antibody, was chosen as an antibody-like protein in the study [20]. The designed anti-SIV immunoadhesin was composed of a single chain antibody (scFv) fused with an Fc portion of immunoglobulin constant domains. The study focused on two immunoadhesins, 4L6 and 5L7, along with an immunoadhesin (N4) that scFv was replaced with the binding domain of rhesus CD4. 

Each cohort of three monkeys was administered intramuscularly with either the serotype 1 of scAAV (scAAV1) for delivery of 4L6 and 5L7 or the serotype 1 of conventional single-stranded AAV (AAV1) at a dose of 2 × 10^13^ gc. The monkeys given VIP to express 4L6 exhibited impressive antibody production, which reached 100–190 μg/mL in 4 weeks post-vector injection, peaked in 4–6 months, and was maintained above 200 μg/mL thereafter. A great variation was seen in the 5L7 cohort, in which two monkeys had serum immunoadhesin of 40 μg/mL and 175 μg/mL at week 4 post-vector injection, while one monkey had 50 μg/mL immunoadhesin at 2 weeks, but lost its expression at week 4 post-vector injection. The N4 cohort had much lower serum concentration of immunoadhesin, presumably from the lower vector-mediated expression by the single-stranded AAV1. All monkeys were challenged intravenously with SIV at week 4 post-vector injection. Remarkably, complete protection was seen in the entire 4L6 cohort, one monkey in the 5L7 cohort, and two monkeys in the N4 cohort. Further analysis revealed that the loss of 5L7 expression by one monkey was caused by the generation of immunoadhesin-specific antibody responses, which might have facilitated the clearance of transgene-expressing cells. These transgene-specific antibody responses were also detected in two other monkeys, one from the 5L7 cohort and one from the N4 cohort, albeit at a much lower level. The analysis of correlation of protection revealed that VIP protected only monkeys without detectable antibody responses against immunoadhesin. Nevertheless, the study by the Johnson team shows that VIP can provide robust protection against virus infection if immune responses against transgenes are appropriately controlled. It also highlights the superiority of natural antibody architecture, which should have lower immune responses as compared to non-self designer proteins, such as immunoadhesin. 

In the same satellite symposium at the AIDS Vaccine 2012 Conference, Dr. Johnson explained how his work had progressed since his last publication. He showed that the monkeys given VIP to express 4L6 immunoadhesin were able to maintain stable expression with serum concentration of ~20 μg/mL for over 5 years. This result is consistent with another previous study, in which intramuscular injection of AAV1 could maintain the expression of erythropoietin protein in monkeys for longer than 6 years [116]. This is exciting news because it means that long-term protection by VIP is possible in primates. Johnson also shared the result on comparing the neutralization activity of the full antibody *vs.* immunoadhesin forms of PG9. The full antibody architecture had neutralization potency that was 10-fold greater than that of immunoadhesin, which corroborates a previous report in a similar study [117]. Because of the potency and immunogenicity, Dr. Johnson concluded in the meeting that the natural antibody architecture is preferred for future VIP testing in humans [23]. 

## 5. Concluding Remarks

Given that the quest for an effective vaccine through active immunization remains elusive, unorthodox approaches, such as VIP by antibody gene transfer, could raise new hopes in combating the ongoing global HIV pandemic. The studies exploring VIP as a novel modality of HIV prophylaxis in mice and macaques show encouraging results. Since the concept has been demonstrated and the preclinical experiments have been completed, the next hurdle is to evaluate such approach in humans. Indeed, two separate phase I trials are in the planning stage to test VIP delivered by intramuscular administration of AAVs encoding antibodies [23]. The trial led by Dr. Johnson and his colleagues is to use AAV1 for the delivery of the PG9 antibody into high-risk, seronegative volunteers. Discussions with the US FDA for the trial plan have been completed, and the clinical-grade vector has been manufactured. The trial is expected to be launched at the end of 2013 or in early 2014. In collaboration with Vaccine Research Center and the U.S. National Institutes of Health, the Baltimore team is planning a clinical trial to test VIP by AAV8-mediated delivery of the VRC01 antibody via muscle injection to volunteers receiving treatment with ART. 

As we anxiously await the outcomes of these two trials, we should also recognize that several barriers lie ahead. First, we do not know how preclinical results in mice and macaques will be translated to humans. Although macaques may be the best animals to model HIV, they can be infected only by SIV and SHIV (chimeric simian/human immunodeficiency virus) [118,119,120], not HIV directly. Thus, it is unclear how the result of the model virus-based challenge experiment conducted in macaques given VIP to express HIV-specific bnAbs can be related to the true HIV control in humans. It seems that human trial remains the best means to determine successful VIP protocols, such as doses, sites of muscle injection, and serotypes of AAVs. Second, since VIP is a form of AAV-based gene therapy, it is subject to all the safety issues involved with this type of gene therapy, such as the immunogenicity of AAV vectors [45] and AAV-mediated insertional mutagenesis [121]. This becomes even more important when one considers that the intention of VIP is to provide prophylaxis for healthy individuals. Encouragingly, recent studies document several therapeutic successes of AAV-based gene therapy for genetic diseases in clinical trials [47,48,49,50,51,52,53,54,56,57,58], and Glybra, which involves the genetic delivery of lipoprotein lipase (LPL) by intramuscular injection of AAV1 vector to treat LPL deficiency disease, has just been licensed in Europe [55]. These successes ease some of the safety concerns and will certainly guide the clinical development of VIP. Finally, constitutive expression could induce immune responses against the VIP antibody and/or the F2A linker, which, in turn, could lead to reduced antibody production and other unknown effects. As a safety precaution, it might be desirable to develop a regulated expression system, such as the dimerizer-regulated gene expression system [116,122,123] to switch antibody expression on and off. 
